# Associations of METS-VF and BRI with risk of osteoarthritis in patients with diabetes or prediabetes: evidence from the NHANES 1999–2020

**DOI:** 10.3389/fendo.2025.1690153

**Published:** 2026-01-30

**Authors:** Wanqing Ye, Lei Wei, Zheng Wang, Yinghu Deng, Zhixiang Ma, Wei Ling, Jun Mei, Yinpeng Dong, Xiaosi Zhang, Chao Miao

**Affiliations:** 1 Department of Orthopedic Trauma and Joint Surgery, Lanxi People’s Hospital, Lanxi, Zhejiang, China; 2 Department of Sports Medicine, Tongling People’s Hospital, Tongling, Anhui, China; 3 Department of Cardiology, The Second People’s Hospital of Hefei, Hefei Hospital Affiliated to Anhui Medical University, Hefei, Anhui, China

**Keywords:** body roundness index, diabetes, NHANES, osteoarthritis, prediabetes, visceral fat metabolism score

## Abstract

**Objective:**

This study aimed to explore the relationship between METS-VF and BRI with OA risk in DM or Pre-DM patients, evaluate their predictive value, and assess the additive effect of their combined application.

**Methods:**

We utilized data from the NHANES 1999-2020, including adult participants diagnosed with diabetes or prediabetes. Multivariable logistic regression was employed to analyze the association between METS-VF and BRI with OA risk, while RCS models were used to explore non-linear relationships. ROC curves were generated to evaluate the predictive value of these indicators for osteoarthritis and determine optimal cut-off values. Based on these cut-offs, participants were divided into four groups to further assess the impact of different combinations on osteoarthritis risk, followed by subgroup analyses. Finally, we analyzed the additive predictive effect of combining METS-VF and BRI.

**Results:**

A total of 2,614 patients with diabetes or prediabetes were included, of whom 450 (17.21%) were diagnosed with osteoarthritis. Multivariable analysis revealed that both METS-VF and BRI were significantly associated with OA risk (METS-VF: OR = 4.02, 95% CI: 2.24-7.19, p<0.0001; BRI: OR = 2.78, 95% CI: 1.60-4.85, p<0.001). RCS analysis demonstrated a non-linear relationship between METS-VF and osteoarthritis risk, while BRI exhibited a linear relationship with OA risk. ROC analysis determined optimal cut-off values of 7.25 for METS-VF and 5.87 for BRI. Compared to the “Low BRI and Low METS-VF” group, the “High BRI and Low METS-VF” group showed an increased risk of OA, although not statistically significant (OR = 1.73, 95% CI: 0.92-3.27, p=0.93). Additive effect analysis found that the combination of BRI and METS-VF (AUC = 0.650) showed no statistical advantage over BRI alone (AUC = 0.632) or METS-VF alone (AUC = 0.650) (DeLong test p>0.05 for both comparisons). Continuous NRI and IDI analyses further confirmed that the combination of BRI and METS-VF did not demonstrate significant additive effects compared to either indicator alone.

**Conclusions:**

METS-VF and BRI are independent predictors of OA risk in patients with diabetes or prediabetes, but their combined application does not enhance predictive value. This suggests that in clinical practice, for OA risk assessment in patients with diabetes or prediabetes, either BRI or METS-VF can be selected individually, without the necessity of evaluating both simultaneously.

## 1 Introduction

Osteoarthritis (OA) is a chronic degenerative joint disease with increasing global prevalence, becoming a leading cause of disability among middle-aged and elderly populations (1). OA represents a complex, whole-joint pathology characterized by progressive degenerative and pathological changes across all articular tissues, including cartilage, subchondral bone, synovium, menisci, ligaments, and infrapatellar fat pad (IFP). Each of these tissues undergoes specific but interconnected degenerative processes that collectively promote disease progression (2, 3). With accelerating population aging and the obesity epidemic, the disease burden of OA is expected to further intensify, posing enormous challenges to healthcare systems and socioeconomic structures (4).

The infrapatellar fat pad (IFP), despite being frequently underrepresented in OA research, plays a significant role as an active local player in OA pathology. The IFP becomes inflamed and fibrotic, and undergoes biomechanical changes affecting OA progression (5). Furthermore, IFP functions as a local source of inflammatory cytokines, which promote joint inflammation and facilitate both peripheral and central sensitization, exacerbating pain in knee OA (6, 7). These inflammatory mediators contribute to cartilage degradation and synovial inflammation, further accelerating the degenerative processes within the joint (8).

OA is a multifactorial disease with several risk factors involved in its onset and progression. These include age, female gender, obesity, genetic predisposition, mechanical load, previous joint injuries, metabolic syndrome, diabetes, hyperlipidemia, and other metabolic disorders (9, 10). Epidemiological studies indicate that patients with Diabetes mellitus (DM) and Prediabetes mellitus (Pre-DM) have significantly higher risk of OA compared to the general population, an association that extends beyond the influence of common risk factors such as age and obesity (11, 12).

The relationship between metabolic disorders (particularly DM/Pre-DM) and OA represents a complex pathophysiological network with multiple interconnected mechanisms. Diabetes creates a systemic metabolic environment characterized by chronic low-grade inflammation, oxidative stress, insulin resistance, and accumulation of advanced glycation end products (AGEs) that directly impacts joint tissues (13). AGEs accumulation in articular cartilage increases its stiffness and brittleness, compromising its biomechanical properties and resistance to mechanical stress. Meanwhile, the chronic inflammatory state activates synoviocytes and chondrocytes to produce matrix-degrading enzymes, accelerating cartilage breakdown. Additionally, hyperglycemia-induced oxidative stress disrupts normal chondrocyte function and promotes cellular senescence, further impairing cartilage repair capacity.

In this complex metabolic-inflammatory environment, accurate assessment of body composition and fat distribution becomes critical, as these factors significantly influence both metabolic health and joint pathology. Traditional anthropometric measures like BMI have limitations in characterizing the metabolic impact of adiposity, as they fail to distinguish between different fat distribution patterns and their metabolic consequences (14, 15). This is particularly relevant in DM/Pre-DM patients, where visceral adiposity and altered fat metabolism play central roles in disease progression and complication development.

Recent studies emphasize that fat distribution patterns have a more critical impact on metabolic health than total fat mass (14). Visceral fat accumulation and dysfunction are considered core links connecting obesity, metabolic diseases, and systemic inflammation (15). The Visceral Fat Metabolism Score (METS-VF), as an emerging composite indicator, integrates anthropometric parameters and blood biochemical markers to accurately reflect visceral fat functional status (16). Compared to traditional visceral fat measurement methods, such as Computed Tomography (CT) or Magnetic Resonance Imaging (MRI), METS-VF offers advantages of being non-invasive, cost-effective, and easily applicable in clinical settings (17). Similarly, the BRI, based on principles of human geometry, evaluates body shape distribution characteristics through the relationship between waist circumference and height, and has been proven to reflect central obesity more accurately than Body mass index (BMI) (18). Previous research has demonstrated that Body Roundness Index (BRI) is closely associated with the risk of various metabolism-related diseases (19).

The interrelationship between METS-VF, BRI, and OA in the context of diabetes and prediabetes represents a critical conceptual framework for understanding metabolic OA pathogenesis. METS-VF captures the functional metabolic status of visceral fat, which serves as a primary source of pro-inflammatory adipokines and cytokines that can directly impact joint tissues. These inflammatory mediators, including TNF-α, IL-1β, and IL-6, propagate systemic low-grade inflammation and directly contribute to cartilage matrix degradation when reaching the joint environment (20). BRI, by characterizing body shape and central adiposity, reflects the mechanical loading component of OA risk while also serving as a proxy for metabolic dysfunction associated with central obesity. In patients with diabetes and prediabetes, these relationships are particularly pronounced, as hyperglycemia amplifies both the inflammatory effects of visceral adiposity (captured by METS-VF) and the metabolic consequences of altered body fat distribution (reflected by BRI).

The clinical significance of understanding these relationships extends beyond academic interest. Current clinical practice lacks effective tools for early identification of high-risk individuals for OA among DM/Pre-DM patients, severely limiting the implementation of targeted prevention and intervention strategies (21). By elucidating how METS-VF and BRI relate to OA risk in the diabetic population, we can potentially develop more effective risk stratification approaches that consider both metabolic and biomechanical components of OA pathogenesis. This would facilitate personalized preventive strategies that simultaneously address hyperglycemia, adiposity-related inflammation, and altered biomechanics, potentially interrupting the vicious cycle that accelerates joint degeneration in this high-risk population.

Despite the promising applications of METS-VF and BRI in assessing metabolic health and body shape distribution, their association with OA risk in DM/Pre-DM patients has not been systematically investigated. Considering that visceral fat dysfunction and altered body shape distribution may be key links connecting metabolic disorders with OA development (20), exploring the value of METS-VF and BRI in OA risk assessment among DM/Pre-DM patients has significant clinical implications. Furthermore, determining optimal cut-off values for these indicators and evaluating the additive effect of their combined application would help construct more precise risk prediction models (22). Research findings based on large representative populations can provide scientific evidence for developing OA prevention strategies targeted at DM/Pre-DM patients, promoting precision medicine practices (23).

Therefore, this study aims to investigate the association between METS-VF and BRI with OA risk in DM/Pre-DM patients based on nationally representative NHANES 1999–2020 data, evaluate their clinical application value as risk prediction tools, and further analyze the potential advantages of their combined application. These findings will address current research gaps, provide clinicians with practical risk assessment tools, and ultimately improve early identification and intervention strategies for OA in DM/Pre-DM patients.

## 2 Methods

### 2.1 Study design and data sources

This study employed a cross-sectional design, utilizing data from eleven consecutive two-year cycles of the NHANES from 1999 to 2020. NHANES (https://wwwn.cdc.gov/nchs/nhanes/default.aspx) is an ongoing, nationwide survey conducted by the National Center for Health Statistics of the Centers for Disease Control and Prevention, designed to assess the health and nutritional status of the United States population. The survey implements a complex, multistage, stratified, probability sampling design, ensuring that the sample is nationally representative across age, gender, race/ethnicity, and income categories.

NHANES encompasses detailed questionnaires, physical examinations, and laboratory tests, providing comprehensive health and nutrition data. All participants provided informed consent, and the survey protocol was approved by the NCHS Research Ethics Review Board. This research utilized publicly available data and adhered to NHANES data use policies and guidelines.

### 2.2 Study subjects

This study included participants aged 20 years and older from the NHANES 1999–2020 survey who met the diagnostic criteria for diabetes or prediabetes. Diabetes was defined based on the following indicators: fasting blood glucose ≥7.0 mmol/L or 2-hour oral glucose tolerance test level ≥11.1 mmol/L; random blood glucose ≥11.1 mmol/L; HbA1c ≥6.5%; use of diabetes medication or insulin; or physician-diagnosed diabetes. Prediabetes was defined by the following criteria: fasting blood glucose between 5.6-7.0 mmol/L or 2-hour oral glucose tolerance test level between 7.8-11.0 mmol/L;HbA1c ≥5.7% and <6.5%; or physician diagnosis of prediabetes (24). They were classified according to BMI as non-obese (BMI < 25 kg/m ²), overweight (BMI 25–30 kg/m ²), and obese (BMI > 30 kg/m ²).

Exclusion criteria comprised: ①missing information on osteoarthritis status; ②missing critical data required for calculating METS-VF or BRI; ③pregnant individuals; and ④missing data for key covariates.

### 2.3 Definition and measurement of variables

#### 2.3.1 Outcome variables: osteoarthritis

The primary outcome variable in this study was OA status, defined as self-reported physician-diagnosed arthritis further confirmed as osteoarthritis (rather than rheumatoid arthritis or other types of arthritis). Participants were categorized into OA and non-OA groups based on self-reported OA status from questionnaire responses. This involved a two-part assessment: ①participants were asked, “Has a doctor or other healthcare professional ever told you that you have arthritis?” Those who answered “yes” proceeded to the next question; ②they were then asked, “What type of arthritis was it?” Subjects who responded “OA” were classified as having osteoarthritis (25).

#### 2.3.2 Exposure variables: METS-VF and BRI

METS-VF was calculated using the equation: METS-VF = 4.466 + 0.011[(Ln(METS-IR))³] + 3.239[(Ln(WHtR))³] + 0.319(Sex) + 0.594(Ln(Age)), where male=1 and female=0. The Metabolic Insulin Resistance score (METS-IR) was derived from the formula: METS-IR = Ln[(2×fasting glucose) + fasting triglycerides)×BMI]/[Ln(high-density lipoprotein cholesterol)]. The waist-to-height ratio (WHtR) was computed as waist circumference divided by height (26).

BRI was calculated according to the formula proposed by Thomas et al.: BRI = 364.2-365.5×(1-[WC(m)/2π]²/[0.5×H(m)]²)½, where Waist circumference(WC) represents waist circumference (m) and H represents height (m) (11). Trained NHANES examiners measured waist circumference and height using standardized methods. Blood samples were collected after a 9-hour fast and analyzed in Centers for Disease Control and Prevention(CDC)-certified laboratories to obtain biochemical indicators including blood glucose, triglycerides, and High density lipoprotein cholesterol(HDL-C).

#### 2.3.3 Covariates

To control for confounding effects, multiple demographic and clinical characteristics were included. Demographic features comprised age, sex, race/ethnicity, education level, poverty income ratio (PIR), and marital status. Lifestyle factors encompassed smoking status (never, former, current smoker) and alcohol consumption (never, former, mild, moderate, heavy drinker). Medical history included hypertension. Functional indicators covered serum creatinine, uric acid (UA), blood urea nitrogen (BUN), and estimated Glomerular Filtration Rate (eGFR). Glucose metabolism was assessed through fasting plasma glucose(FPG) and hemoglobin A1c(HbA1c). Physical activity was evaluated based on work activity and recreational activity levels.

### 2.4 Subgroup analysis by diabetes status

Following the primary analyses, we performed stratified analyses to separately evaluate the associations of METS-VF and BRI with OA risk in patients with diabetes and those with prediabetes. Participants were categorized based on the previously described diagnostic criteria for diabetes and prediabetes. For each subgroup, we applied the same multivariable logistic regression models (Model 1: unadjusted; Model 2: adjusted for age, sex, race/ethnicity; Model 3: fully adjusted for all covariates) to examine the independent associations of BRI and METS-VF with OA risk. We calculated odds ratios (ORs) with 95% confidence intervals (CIs) and assessed potential effect modification by testing for interaction between diabetes status and the main exposure variables (METS-VF and BRI).

### 2.5 Statistical analysis

All analyses accounted for NHANES complex sampling design, utilizing sampling weights. Participants were categorized into OA and non-OA groups based on osteoarthritis occurrence. Continuous variables were presented as means (standard errors), while categorical variables were expressed as percentages (standard errors). Between-group differences were compared using t-tests, Mann-Whitney U tests, chi-square tests, or Fisher’s exact tests. Multivariable logistic regression models assessed the independent associations of BRI and METS-VF with OA risk. Three progressively adjusted models were constructed: Model 1 (unadjusted); Model 2 (adjusted for age, sex, race/ethnicity); and Model 3 (further adjusted for BUN, eGFR, HbA1c, hypertension, smoking, alcohol consumption, work activity, and recreational activity). Results were presented as OR with 95%CI. RCS analysis examined potential non-linear relationships between BRI, METS-VF, and OA risk. ROC curves evaluated the predictive value of these indices for osteoarthritis and determined optimal cut-off values.

The additive effect of combining BRI and METS-VF for OA risk prediction was assessed through:①Model construction: single-indicator models for BRI and METS-VF; combined BRI+METS-VF model;②Predictive performance evaluation: calculating area under the ROC curve for each model; comparing AUC differences between models using DeLong tests; computing Net Reclassification Improvement(NRI) and Integrated Discrimination Improvement(IDI) to evaluate improvement magnitude. Potential interactive effects between BRI, METS-VF and age, sex, race, and hypertension were examined by incorporating interaction terms into the models, with subsequent subgroup analyses. All statistical analyses were performed using R software (version 4.3.2), with P<0.05 (two-sided) considered statistically significant.

## 3 Results

### 3.1 Basic characteristics of study subjects

**Figure 1:**
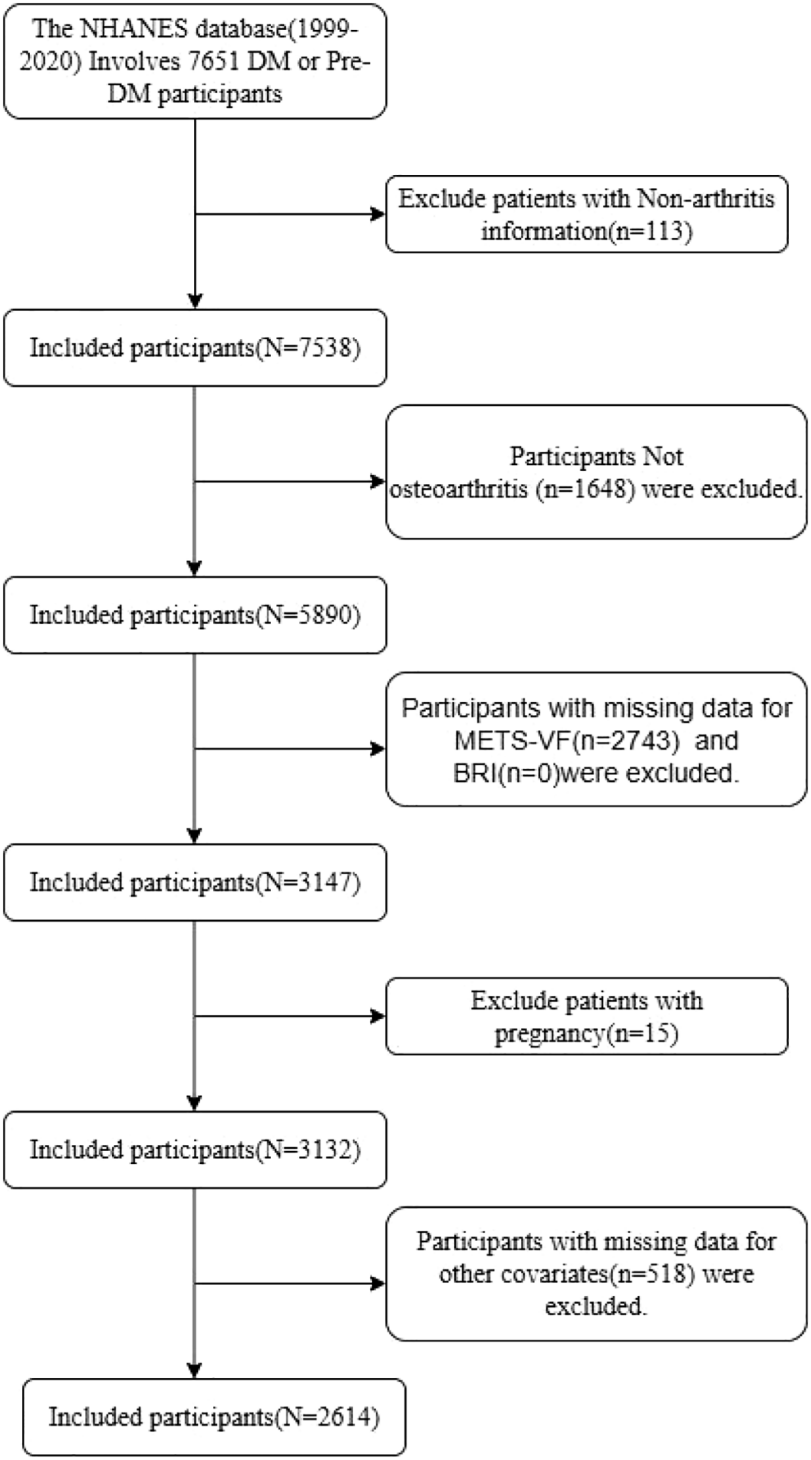
Study flow chart.

A total of 2614 patients with diabetes or prediabetes were included in the study, 450 (17.21%) of whom were diagnosed with osteoarthritis. Detailed study flow was shown in **Figure 1**. Who were divided into two groups based on the presence of OA for comparison. Results revealed that patients in the OA group were significantly older than those in the non-OA group (61.13 ± 0.72 years vs. 48.33 ± 0.50 years, P<0.0001), with a notably higher proportion of females (60.84% vs. 41.12%, P<0.0001) and non-Hispanic whites (80.78% vs. 64.08%, P<0.0001).Regarding clinical characteristics, patients in the OA group exhibited significantly higher BUN levels compared to the non-OA group (5.88 ± 0.16 vs. 4.98 ± 0.05, P<0.0001), while eGFR levels were significantly lower (81.94 ± 1.25 vs. 94.63 ± 0.63, P<0.0001). Most importantly, both BRI and METS-VF were significantly higher in the OA group than in the non-OA group (BRI: 6.61 ± 0.17 vs. 5.70 ± 0.07, P<0.0001; METS-VF: 7.26 ± 0.04 vs. 6.98 ± 0.02, P<0.0001). The prevalence of hypertension was significantly higher in the OA group (60.78% vs. 42.20%, P<0.0001), as was the proportion of obese patients with BMI>30 (54.20% vs. 43.23%, P = 0.01). Regarding lifestyle factors, the OA group had a higher percentage of former smokers (35.44% vs. 26.08%, P = 0.04) and generally lower physical activity levels. Notably, the prevalence of OA showed a clear increasing trend with higher quartiles of BRI and METS-VF. In the fourth quartile groups of BRI and METS-VF, the proportions of OA patients reached 35.73% and 38.74%, respectively, compared to only 14.25% and 11.83% in the first quartile groups (both P<0.0001), suggesting that these two indices may be closely associated with OA risk (**Table 1**).

**Table 1:** OA Patients versus non-OA patients general clinical characteristics.

Variables	Total(n=2614)	Non-OA(n=2164)	OA(n=450)	P-value
Age, mean (SE)	50.77(0.46)	48.33(0.50)	61.13(0.72)	< 0.0001
Creatinine, mean (SE)	78.67(0.56)	78.43(0.64)	79.67(1.50)	0.47
UA, mean (SE)	337.76(2.64)	338.95(2.69)	332.66(6.54)	0.35
BUN, mean (SE)	5.15(0.05)	4.98(0.05)	5.88(0.16)	< 0.0001
PIR, mean (SE)	3.05(0.06)	3.02(0.05)	3.16(0.13)	0.26
eGFR, mean (SE)	92.22(0.55)	94.63(0.63)	81.94(1.25)	< 0.0001
HbA1c, mean (SE)	5.87(0.03)	5.85(0.03)	5.96(0.06)	0.09
BRI, mean (SE)	5.87(0.07)	5.70(0.07)	6.61(0.17)	< 0.0001
METS-VF, mean (SE)	7.03(0.02)	6.98(0.02)	7.26(0.04)	< 0.0001
FPG, mean (SE)	6.45(0.06)	6.44(0.06)	6.50(0.10)	0.6
Sex,%(SE)				< 0.0001
Female	44.87(0.02)	41.12(1.63)	60.84(3.55)	
Male	55.13(0.02)	58.88(1.63)	39.16(3.55)	
Race,%(SE)				< 0.0001
Mexican American	10.26(0.01)	11.88(1.12)	3.33(0.65)	
Non-Hispanic Black	9.56(0.01)	10.38(0.74)	6.09(0.94)	
Non-Hispanic White	67.25(0.03)	64.08(1.50)	80.78(2.30)	
Other	12.93(0.01)	13.67(0.82)	9.81(1.92)	
Marital,%(SE)				< 0.001
Divorced	10.43(0.01)	10.09(1.12)	11.87(2.61)	
Married	59.27(0.02)	58.07(1.52)	64.39(3.78)	
Never married	12.89(0.01)	14.99(1.02)	3.95(0.97)	
Other	17.41(0.01)	16.85(1.13)	19.79(2.84)	
Education,%(SE)				0.8
High school or equivalent	26.00(0.01)	25.95(1.54)	26.22(3.11)	
Less than high school	14.97(0.01)	15.28(0.92)	13.66(2.11)	
Some college or above	59.03(0.03)	58.77(1.85)	60.13(3.51)	
Smoke,%(SE)				0.04
Former	27.86(0.02)	26.08(1.56)	35.44(3.57)	
Never	55.01(0.02)	56.57(1.79)	48.38(3.88)	
Now	17.12(0.01)	17.35(1.01)	16.18(2.38)	
Alcohol,%(SE)				< 0.001
Former	15.29(0.01)	14.87(1.01)	17.08(2.03)	
Heavy	21.71(0.01)	24.03(1.38)	11.79(2.09)	
Mild	38.14(0.02)	36.66(1.46)	44.47(3.48)	
Moderate	14.92(0.01)	14.27(1.05)	17.67(2.93)	
Never	9.94(0.01)	10.17(0.86)	8.99(1.87)	
Hypertension,%(SE)				< 0.0001
No	54.27(0.02)	57.80(1.53)	39.22(3.59)	
Yes	45.73(0.02)	42.20(1.53)	60.78(3.59)	
BMI,%(SE)				0.01
<25	20.38(0.01)	21.79(1.15)	14.39(2.50)	
>30	45.32(0.02)	43.23(1.53)	54.20(3.63)	
25-30	34.30(0.02)	34.98(1.48)	31.41(3.09)	
BRIQ,%(SE)				< 0.0001
Q1	25.81(0.01)	28.52(1.38)	14.25(2.58)	
Q2	25.23(0.02)	26.36(1.52)	20.41(2.60)	
Q3	24.59(0.01)	23.41(1.17)	29.61(3.44)	
Q4	24.38(0.01)	21.72(1.20)	35.73(3.53)	
METS-VFQ,%(SE)				< 0.0001
Q1	27.98(0.02)	31.76(1.46)	11.83(2.52)	
Q2	24.35(0.01)	24.67(1.44)	22.97(3.08)	
Q3	24.26(0.01)	23.75(1.21)	26.46(2.71)	
Q4	23.41(0.01)	19.82(1.22)	38.74(3.31)	
Work activity,%(SE)				0.03
Both	19.38(0.01)	20.51(1.21)	14.56(2.18)	
Moderate	25.33(0.02)	24.49(1.38)	28.90(2.77)	
No	51.18(0.02)	50.39(1.40)	54.52(3.14)	
Vigorous	4.11(0.01)	4.61(0.75)	2.02(0.94)	
Recreational activity,%(SE)				0.004
Both	14.11(0.01)	15.42(1.06)	8.49(2.03)	
Moderate	31.33(0.02)	30.19(1.58)	36.18(3.16)	
No	46.72(0.02)	45.63(1.61)	51.36(2.98)	
Vigorous	7.85(0.01)	8.76(0.96)	3.98(1.37)	

Date are presented as mean (SE) or % (SE); UA, Uric acid; BUN, Blood urea nitrogen; PIR, Poverty income ratio; eGFR, Estimated glomerular filtration rate; HbA1c, Glycosylated haemoglobin; BRI, Body Roundness Index; METS-VF, Visceral Fat Metabolism Score; FPG, Fasting plasm glucose; BMI, Body mass index.

### 3.2 Association analysis of BRI, METS-VF and OA risk

**Tables 2** and **3** present the results of multivariate logistic regression analyses examining the associations between BRI, METS-VF, and OA risk. In the unadjusted model, each unit increase in BRI was associated with a 17% increased risk of OA (OR = 1.17, 95%CI: 1.11-1.23, P<0.0001), while each unit increase in METS-VF was associated with a 190% increased risk (OR = 2.90, 95%CI: 1.99-4.23, P<0.0001). In the fully adjusted model (Model 3), each unit increase in BRI was associated with a 15% increased risk of OA (OR = 1.15, 95%CI: 1.07-1.24, P<0.001), and each unit increase in METS-VF was associated with a 133% increased risk (OR = 2.33, 95%CI: 1.48-3.68, P<0.001). These findings indicate that both BRI and METS-VF are independent risk factors for OA in patients with diabetes or prediabetes. Further analysis with BRI and METS-VF categorized into quartiles (Q1-Q4) revealed a clear dose-response relationship. Compared to the lowest quartile (Q1), the highest quartile (Q4) of BRI was associated with a 178% increased risk of OA (OR = 2.78, 95%CI: 1.60-4.85, P<0.001, Ptrend<0.0001), while the highest quartile of METS-VF was associated with a 302% increased risk (OR = 4.02, 95%CI: 2.24-7.19, P<0.0001, Ptrend<0.0001).

**Table 2:** Association between BRI and risk of OA.

Variables	Model 1		Model 2		Model 3	
	OR(95%CI)	P	OR(95%CI)	P	OR(95%CI)	P
BRI	1.17(1.11,1.23)	<0.0001	1.14(1.06,1.23)	<0.001	1.15(1.07,1.24)	<0.001
BRI Q						
Q1	Ref	Ref	Ref	Ref	Ref	Ref
Q2	1.55(0.93,2.57)	0.09	1.37(0.79,2.39)	0.26	1.33(0.74,2.38)	0.33
Q3	2.53(1.49,4.29)	<0.001	2.19(1.24,3.87)	0.01	2.22(1.25,3.94)	0.01
Q4	3.29(2.05,5.30)	<0.0001	2.68(1.55,4.66)	<0.001	2.78(1.60,4.85)	<0.001
P for trend	<0.0001		<0.001		<0.0001	

OR, Odds ratio; CI, confidence interval; Ref, reference; BRI, Body Roundness Index

Model 1: No adjustments made;

Model 2: Adjusted for Age, Sex, Race;

Model 3: Adjusted for Age, Sex, Race, BUN, eGFR, HbA1c, Hypertension, Smoke, Alcohol, Work activity, Recreational activity

**Table 3:** Association between METS-VF and risk of OA.

Variables	Model 1		Model 2		Model 3	
	OR(95%CI)	P	OR(95%CI)	P	OR(95%CI)	P
METS-VF	2.90(1.99,4.23)	<0.0001	2.21(1.43,3.42)	<0.001	2.33(1.48,3.68)	<0.001
METS-VF Q						
Q1	Ref	Ref	Ref	Ref	Ref	Ref
Q2	2.50(1.41,4.43)	0.002	2.34(1.29,4.26)	0.01	2.33(1.28,4.25)	0.01
Q3	2.99(1.78,5.02)	<0.0001	2.64(1.49,4.66)	<0.001	2.67(1.47,4.85)	0.002
Q4	5.25(3.07,8.98)	<0.0001	3.72(2.06,6.73)	<0.0001	4.02(2.24,7.19)	<0.0001
P for trend	<0.0001		<0.0001		<0.0001	

OR, Odds ratio; CI, confidence interval; Ref, reference; METS-VF, Visceral Fat Metabolism Score

Model 1: No adjustments made;

Model 2: Adjusted for Age, Sex, Race;

Model 3: Adjusted for Age, Sex, Race, BUN, eGFR, HbA1c, Hypertension, Smoke, Alcohol, Work activity, Recreational activity

### 3.3 RCS analysis of BRI, METS-VF and OA risk

**Figure 2:**
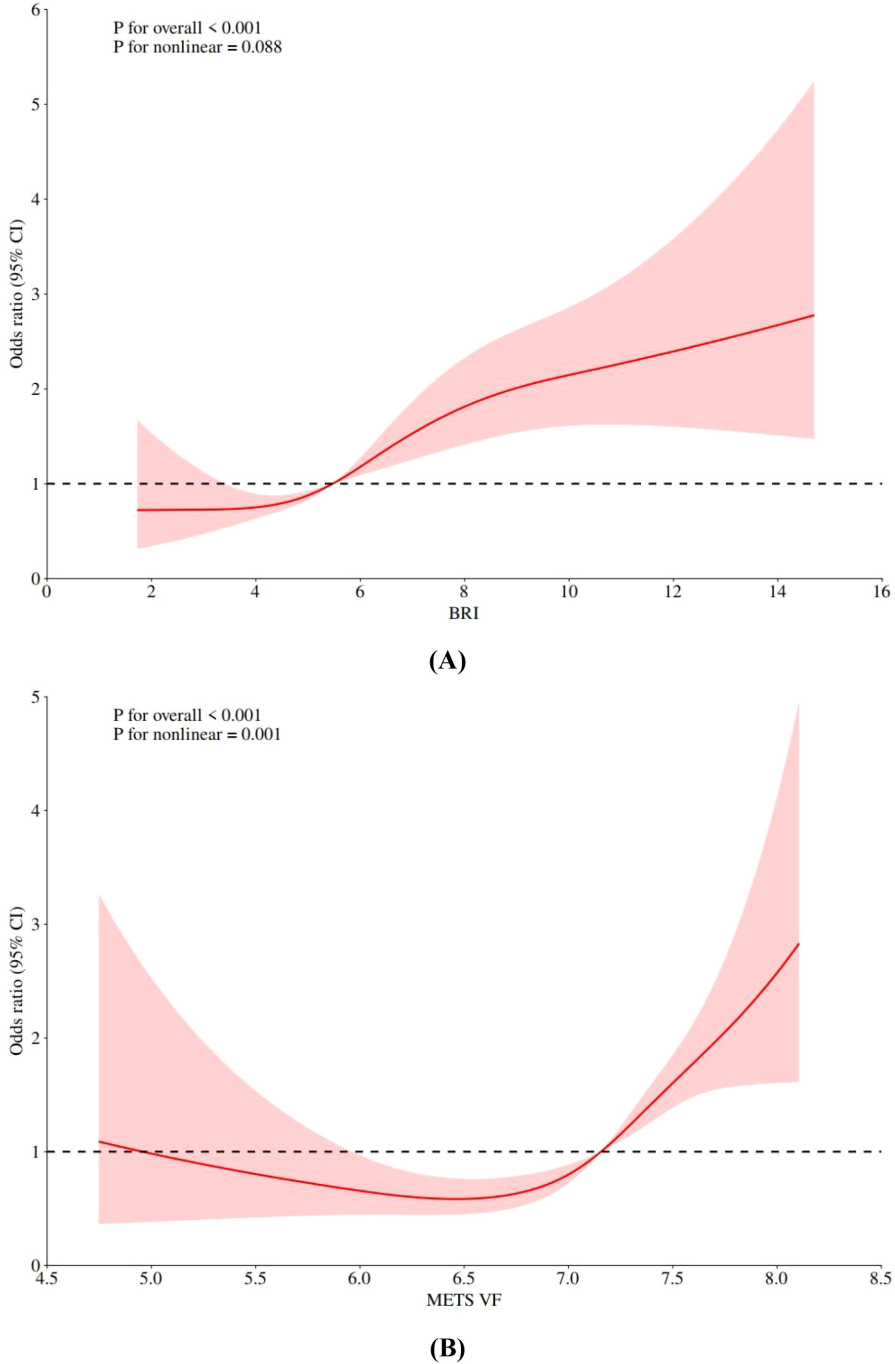
Nonlinear association RCS analysis of BRI, METS-VF and risk of OA in diabetes or prediabetes patients; **(A)** BRI, **(B)** METS-VF; BRI, body roundness index; METS-VF, Visceral Fat Metabolism Score.

**Figure 2** illustrates the dose-response relationship between BRI, METS-VF, and OA risk using RCS analysis. For BRI, the test for non-linearity was not significant (Pnon-linearity=0.088), indicating a linear dose-response relationship between BRI and OA risk. The association between METS-VF and OA risk demonstrated a significant non-linear relationship (P = 0.001).

### 3.4 Additive effect analysis of BRI and METS-VF on OA risk prediction

**Figure 3:**
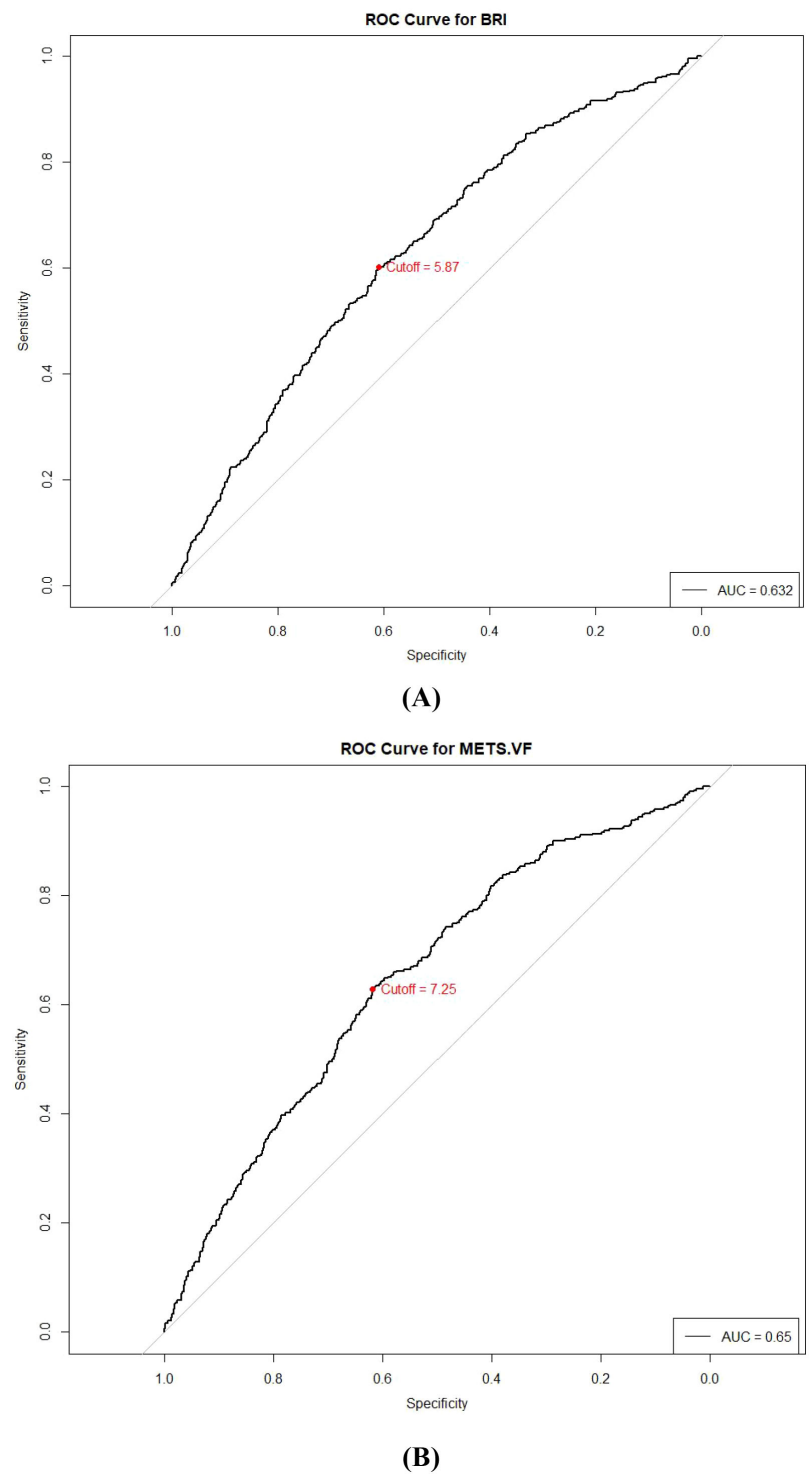
ROC curves were used to assess the value of BRI, METS-VF in predicting osteoarthritis and to determine the optimal cutoff value. **(A)** BRI, **(B)** METS-VF; BRI, Body Roundness Index; METS-VF, Visceral Fat Metabolism Score.

Among all participants, ROC curve analysis was employed to evaluate the predictive value of BRI and METS-VF for osteoarthritis and determine optimal cutoff values, which were identified as 5.865 for BRI and 7.255 for METS-VF (**Figure 3**). **Table 4** presents the results of the additive effect analysis of BRI and METS-VF for OA risk prediction, revealing that patients with high BRI (≥5.865) and low METS-VF (<7.255) had the highest OA risk. In the unadjusted Model 1, patients with high BRI (≥5.865) and low METS-VF (<7.255) showed an OR of 1.41 (95% CI: 0.86-2.32, P = 0.58). Compared to the unadjusted model, the values in the adjusted Model 2 (OR = 1.63, 95% CI: 0.90-2.94, P = 0.55) and Model 3 (OR = 1.73, 95% CI: 0.92-3.27, P = 0.93) showed slight changes but remained statistically non-significant.

**Table 4:** Association of BRI+METS-VF combination with OA in the overall population and various subgroups.

	Low BRI and low METS.VF(BRI<5.865 and METS-VF<7.255)	Low BRI and high METS.VF(BRI<5.865 and METS-VF≥7.255)	High BRI and low METS.VF(BRI≥5.865 and METS-VF<7.255)	High BRI and high METS.VF(BRI≥5.865 and METS-VF≥7.255)	P-value
Overall					
Model 1	Ref	0.66(0.39,1.10)	1.41(0.86,2.32)	1.02(0.72,1.45)	0.58
Model 2	Ref	0.61(0.36,1.01)	1.63(0.90,2.94)	1.07(0.77,1.48)	0.55
Model 3	Ref	0.66(0.40,1.10)	1.73(0.92,3.27)	1.23(0.91,1.67)	0.93
Male					
Model 1	Ref	0.46(0.20,1.03)	1.36(0.67,2.77)	0.63(0.38,1.04)	0.03
Model 2	Ref	0.46(0.19,1.14)	1.59(0.76,3.34)	0.76(0.44,1.30)	0.09
Model 3	Ref	0.57(0.23,1.39)	1.98(0.89,4.40)	1.03(0.62,1.71)	0.30
Female					
Model 1	Ref	0.75(0.39,1.42)	1.57(0.71,3.46)	1.32(0.87,2.01)	0.51
Model 2	Ref	0.75(0.40,1.43)	1.65(0.63,4.33)	1.37(0.89,2.10)	0.48
Model 3	Ref	0.74(0.38,1.42)	1.63(0.58,4.59)	1.31(0.85,2.02)	0.69
Age<60					
Model 1	Ref	0.45(0.15,1.32)	1.18(0.51,2.74)	0.68(0.39,1.17)	0.07
Model 2	Ref	0.40(0.14,1.12)	1.55(0.68,3.55)	0.66(0.38,1.14)	0.03
Model 3	Ref	0.50(0.17,1.44)	1.68(0.73,3.86)	0.70(0.41,1.21)	0.04
Age≥60					
Model 1	Ref	0.77(0.42,1.43)	1.70(0.86,3.35)	1.49(0.97,2.28)	0.36
Model 2	Ref	0.80(0.42,1.51)	1.59(0.72,3.49)	1.38(0.93,2.06)	0.47
Model 3	Ref	0.76(0.39,1.49)	1.52(0.63,3.66)	1.58(1.03,2.41)	0.29
Non-Hispanic White					
Model 1	Ref	0.53(0.24,1.20)	2.00(0.98,4.07)	1.17(0.73,1.87)	0.75
Model 2	Ref	0.50(0.22,1.12)	1.89(0.80,4.46)	1.20(0.77,1.86)	0.68
Model 3	Ref	0.58(0.25,1.35)	2.14(0.81,5.64)	1.49(0.98,2.27)	0.20
Non-Hispanic Black					
Model 1	Ref	0.39(0.12,1.25)	0.92(0.28,2.96)	0.77(0.34,1.76)	0.40
Model 2	Ref	0.42(0.12,1.44)	1.05(0.28,3.91)	0.89(0.38,2.11)	0.62
Model 3	Ref	0.32(0.08,1.28)	0.75(0.19,2.93)	1.23(0.54,2.78)	0.94
Mexican American					
Model 1	Ref	0.56(0.13,2.49)	1.89(0.33,10.78)	1.02(0.33,3.19)	0.68
Model 2	Ref	0.30(0.06,1.48)	1.68(0.40,7.14)	0.70(0.21,2.40)	0.35
Model 3	Ref	0.30(0.04,2.08)	2.60(0.46,14.63)	1.42(0.43,4.62)	0.70
Other Races					
Model 1	Ref	2.84(1.02,7.94)	1.23(0.29,5.25)	1.12(0.65,1.95)	0.41
Model 2	Ref	2.07(0.87,4.91)	1.60(0.33,7.60)	1.04(0.56,1.93)	0.72
Model 3	Ref	2.43(1.01,5.82)	1.73(0.37,8.13)	1.01(0.46,2.22)	0.72
Hypertension					
Model 1	Ref	0.97(0.44,2.13)	1.54(0.80,2.94)	1.16(0.75,1.80)	0.93
Model 2	Ref	0.96(0.44,2.11)	1.97(0.94,4.13)	1.24(0.79,1.93)	0.86
Model 3	Ref	1.00(0.47,2.14)	2.18(1.07,4.43)	1.45(0.96,2.19)	0.80
Non-hypertension					
Model 1	Ref	0.53(0.27,1.03)	1.48(0.62,3.51)	1.01(0.61,1.68)	0.66
Model 2	Ref	0.47(0.25,0.91)	1.50(0.51,4.44)	1.02(0.63,1.66)	0.59
Model 3	Ref	0.55(0.29,1.06)	1.14(0.34,3.80)	1.01(0.60,1.67)	0.61

METS-VF, Visceral Fat Metabolism Score; BRI, Body Roundness Index.

Model 1: No adjustments made;

Model 2: Adjusted for Age, Sex, Race;

Model 3: Adjusted for Age, Sex, Race, BUN, eGFR, HbA1c, Hypertension, Smoke, Alcohol, Work activity, Recreational activity

**Figure 4:**
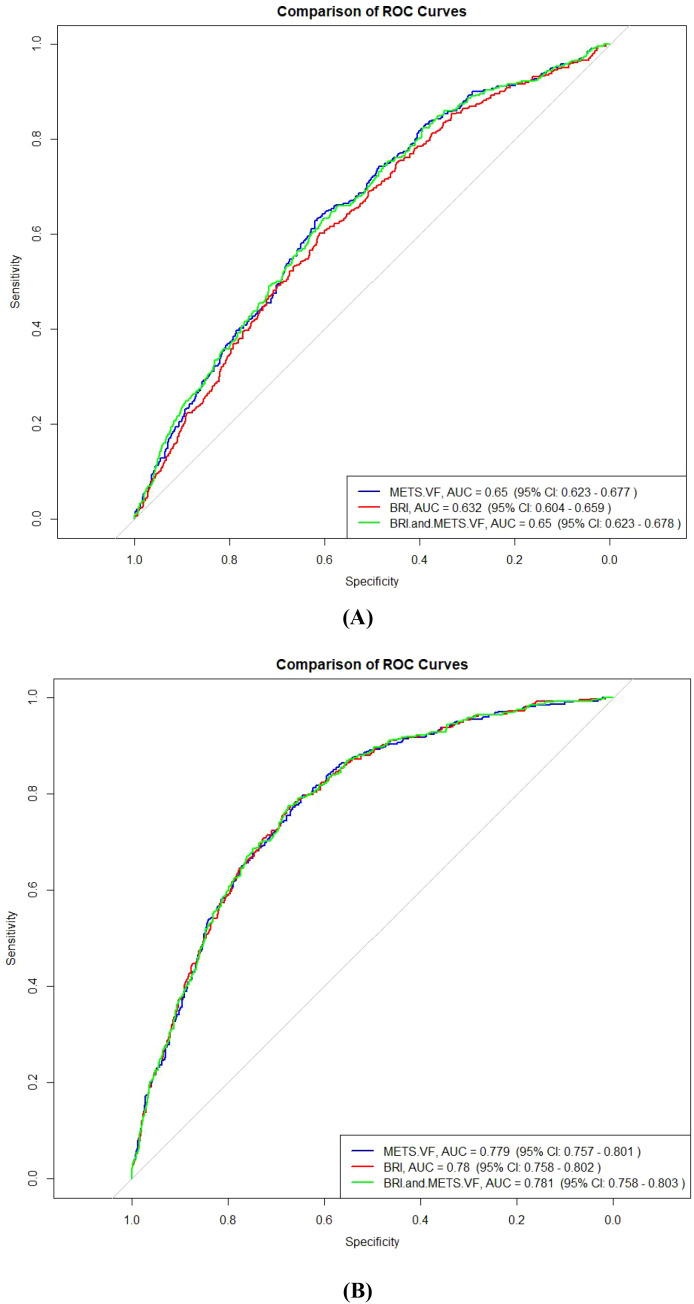
Comparison of additive effect between BR and METS-VF on OA risk prediction by ROC analysis. **(A)** Unadjusted covariates, **(B)** Covariate-adjusted ROC curve analysis. BRI, Body Roundness Index; METS-VF, Visceral Fat Metabolism Score.

**Table 5** and **Figure 4** compare the area under the ROC curve for different models: BRI: AUC = 0.632 (95% CI: 0.604-0.659); METS-VF: AUC = 0.650 (95% CI: 0.623-0.677); and BRI+METS-VF: AUC = 0.650 (95% CI: 0.623-0.678). DeLong test results indicated that the combined BRI and METS-VF model (AUC = 0.650) did not significantly improve the predictive ability for OA risk compared to BRI (AUC = 0.632) or METS-VF (AUC = 0.650) alone (P>0.05).

**Table 5:** Predictive value of BRI, METS-VF and their combination for risk of OA in patients with diabetes or prediabetes.

	Harrell’s C-index	P-value	Adjusted Harrell’s C-index^*^	P-value
BRI vs. METS.VF	0.632(0.604-0.659) vs. 0.650(0.623-0.677)	0.0114	0.780(0.758-0.802) vs. 0.779(0.757-0.801)	0.5577
BRI+METS.VF vs. BRI	0.650(0.623-0.678) Vs. 0.632(0.604-0.659)	1.0000	0.781(0.758-0.803) vs. 0.780(0.758-0.802)	1.0000
BRI+METS.VF vs. METS.VF	0.650(0.623-0.678) Vs. 0.650(0.623-0.677)	0.9765	0.781(0.758-0.803) vs. 0.779(0.757-0.801)	0.2970

METS-VF, Visceral Fat Metabolism Score; BRI, Body Roundness Index.^*^: Covariate-adjusted receiver operating characteristic curve analysis (Adjusted for Age, Sex, Race, BUN, eGFR, HbA1c, Hypertension, Smoke, Alcohol, Work activity, Recreational activity).

Additionally, we calculated the NRI and IDI to assess the additive predictive effect. Compared to the BRI model, the combined BRI+METS-VF model showed a continuous NRI of 0.219 (95% CI: 0.116-0.322, P<0.0001) and an IDI of 0.015 (95% CI: 0.010-0.020, P<0.0001), indicating improvement in both NRI and IDI. However, compared to the METS-VF model, the combined BRI+METS-VF model demonstrated a continuous NRI of 0.003 (95% CI: 0.001-0.005, P = 0.004) and an IDI of 0.076 (95% CI: -0.026-0.174, P = 0.156), suggesting that the improvement in NRI was not substantial and the IDI did not reach statistical significance (**Table 6**).

**Table 6:** Comparison of NRI and IDI for predictive value of BRI, METS-VF and their combination for risk of OA in diabetes or prediabetes patients.

Models	AUC(95%CI)	NRI (Continuous)	P-value	IDI	P-value
BRI	0.632(0.604-0.659)	–	–	–	–
METS-VF	0.650(0.623-0.677)	–	–	–	–
BRI+METS-VF^#^	0.650(0.623-0.678)	0.219(0.116-0.322)	<0.0001	0.015(0.010-0.020)	<0.0001
BRI+METS-VF^*^	0.650(0.623-0.678)	0.003(0.001-0.005)	0.004	0.076(-0.026-0.174)	0.156

^#^: BRI+METS-VF vs. BRI;^*^: BRI+METS-VF vs. METS-VF; METS-VF: Visceral Fat Metabolism Score, BRI: Body Roundness Index

### 3.5 Subgroup analyses by diabetes status

To provide more specific insights for targeted clinical interventions, we performed separate analyses for patients with diabetes (n=1,173) and those with prediabetes (n=1,441). The prevalence of OA was higher in the diabetes group (19.78%, n=232) compared to the prediabetes group (15.13%, n=218), although this difference was not statistically significant after adjusting for age and other demographic factors (p=0.09). **Table 7** presents the results of multivariable logistic regression analyses for the diabetes and prediabetes subgroups. In patients with diabetes, each unit increase in BRI was associated with a 20% increased risk of OA in the fully adjusted model (OR = 1.20, 95% CI: 1.09-1.33, P<0.001), while each unit increase in METS-VF was associated with a 158% increased risk (OR = 2.58, 95% CI: 1.35-4.93, P<0.01). Similarly, in patients with prediabetes, BRI (OR = 1.11, 95% CI: 1.01-1.22, P = 0.03) and METS-VF (OR = 2.11, 95% CI: 1.14-3.90, P = 0.02) were both significantly associated with OA risk, although the magnitudes of these associations were somewhat weaker than in the diabetes subgroup. The interaction between diabetes status and METS-VF was statistically significant (Pinteraction=0.03), suggesting that the association between METS-VF and OA risk was stronger in patients with diabetes than in those with prediabetes. No significant interaction was found between diabetes status and BRI (Pinteraction=0.09).

**Table 7:** Subgroup analyses of the association between BRI, METS-VF and OA risk by diabetes status.

Subgroup	Exposure	Model 1		Model 2		Model 3	
		OR(95%CI)	P	OR(95%CI)	P	OR(95%CI)	P
Diabetes(n=1173)	BRI	1.28 (1.18-1.39)	<0.001	1.24 (1.13-1.36)	<0.001	1.20 (1.09-1.33)	<0.001
	METS-VF	2.97 (1.63-5.42)	<0.001	2.79 (1.51-5.17)	<0.001	2.58 (1.35-4.93)	<0.01
Prediabetes(n=1441)	BRI	1.19 (1.09-1.30)	<0.001	1.15 (1.05-1.26)	<0.01	1.11 (1.01-1.22)	<0.01
	METS-VF	2.46 (1.39-4.35)	<0.01	2.27 (1.26-4.08)	<0.01	2.11 (1.14-3.90)	<0.05
Interaction P-value	BRI × diabetes status	0.06		0.07		0.09	
	METS-VF × diabetes status	0.02		0.02		0.03	

OR, Odds ratio; CI, confidence interval; Ref, reference; METS-VF, Visceral Fat Metabolism Score

Model 1: No adjustments made;

Model 2: Adjusted for Age, Sex, Race;

Model 3: Adjusted for Age, Sex, Race, BUN, eGFR, HbA1c, Hypertension, Smoke, Alcohol, Work activity, Recreational activity

## 4 Discussion

This study is the first systematic investigation of the associations between METS-VF, BRI, and the risk of OA in patients with diabetes or prediabetes. Based on extensive representative data from the NHANES 1999-2020, we found that both METS-VF and BRI are independent predictors of OA risk in patients with diabetes or prediabetes, with both showing significant dose-response relationships. These findings not only expand the current understanding of the relationship between metabolic factors and OA but also provide new perspectives and tools for clinical risk assessment and early intervention.

Our research demonstrates that, after adjusting for multiple potential confounding factors, each unit increase in BRI was associated with a 15% increased risk of OA, while each unit increase in METS-VF was associated with a 133% increased risk. This strong independent association highlights the important role of fat distribution patterns and metabolic health status in OA pathogenesis. Niu et al.’s findings from the Framingham OA study are highly consistent with our results, reporting that central obesity was significantly associated with the risk of knee OA progression, independent of BMI (27). Furthermore, in a prospective study of the Southern Swedish Health cohort, Lohmander et al. found that waist circumference was positively associated with the risk of severe OA (requiring joint replacement surgery), and this association persisted after adjusting for BMI (28). Our study further extends these findings by specifically focusing on the particular high-risk population of patients with diabetes or prediabetes, a group that is especially important in clinical practice but has received less targeted attention in previous research. Unlike previous studies, we employed more comprehensive and clinically practical indices for assessing metabolic health and body shape distribution. BRI, as a geometric index based on waist circumference and height, more accurately reflects central obesity compared to BMI (29). Meanwhile, METS-VF, as an integrated score incorporating multiple anthropometric and metabolic parameters, may more comprehensively reflect visceral fat functional status than traditional single indicators like waist circumference or waist-to-hip ratio (17). This provides clinicians with more convenient and integrative risk assessment tools.

Through quartile analysis and RCS analysis, this study thoroughly investigated the dose-response relationship between METS-VF, BRI, and OA risk. The quartile analysis revealed a clear stepwise risk increase trend: compared to the lowest quartile, the highest quartile of BRI was associated with a 178% increased risk of OA, while the highest quartile of METS-VF was associated with a 302% increased risk. This significant dose-response relationship further strengthens the reliability and biological plausibility of their associations with OA risk. Notably, RCS analysis revealed a significant non-linear relationship between METS-VF and OA risk (P = 0.001), while BRI demonstrated a linear association (Pnon-linearity=0.088). This finding echoes the results of Collins et al., who identified complex non-linear interactions between metabolic syndrome components and knee OA (30). The concept of a “metabolic joint inflammation threshold” proposed by Schett et al. may provide a theoretical explanation, suggesting that metabolic inflammation might accelerate joint tissue damage beyond a specific threshold (31). As a comprehensive metabolic indicator, METS-VF may more sensitively capture this threshold effect, while BRI, primarily reflecting body shape distribution, may maintain a relatively linear relationship with OA risk. Rosa et al. observed similar non-linear patterns in a cross-sectional study on the relationship between metabolic syndrome and knee OA, speculating that this might be related to the differential effects of various metabolic disorder combinations on the joint metabolic microenvironment (32). Our findings not only align with this speculation but also provide more refined quantitative evidence, indicating that when assessing and managing OA risk in patients with diabetes or prediabetes, the non-linear impact of metabolic indicators must be considered.

The optimal cutoff values for BRI and METS-VF determined through ROC curve analysis in this study were 5.865 and 7.255, respectively, providing objective standards for identifying high-risk individuals in clinical practice. These cutoff values have important clinical translational value, helping healthcare professionals easily screen for high-risk patients who may require more aggressive intervention. Compared to the cutoff values for body fat distribution indicators determined by Dawson et al. in European populations, our results show certain differences (33). This discrepancy may reflect racial differences and the influence of specific disease backgrounds (diabetes/prediabetes). Cheon et al.’s research suggests that Asian populations may require specific body fat distribution indicator cutoff values to accurately assess metabolic risk (34), which aligns with our findings and emphasizes the necessity of validating these cutoff values in populations with different racial and disease backgrounds. Our study specifically determined the optimal cutoff values for METS-VF and BRI in the particular high-risk population of patients with diabetes or prediabetes, filling an important knowledge gap in this field and supporting precision medicine practices.

Surprisingly, our additive effect analysis showed mixed results when evaluating the combined application of BRI and METS-VF for OA risk prediction. When assessed by the conventional discrimination metric (AUC), the combined BRI and METS-VF model (AUC = 0.650) did not significantly improve the predictive ability for OA risk compared to BRI (AUC = 0.632) or METS-VF (AUC = 0.650) alone (P>0.05 for both comparisons by DeLong test).This is not entirely consistent with Berenbaum et al.’s hypothesis, which suggested that integrating multiple metabolic indicators should more accurately predict OA risk (35). However, further analysis using more sensitive measures of predictive improvement revealed a more nuanced pattern. Compared to BRI alone, the combined BRI+METS-VF model showed a significant improvement in risk classification, with a continuous NRI of 0.219 (95%CI: 0.116-0.322, P<0.0001) and an IDI of 0.015 (95%CI: 0.010-0.020, P<0.0001). This indicates that while overall discriminative ability was not significantly enhanced, the combined model did provide meaningful improvements in correctly classifying individuals’ risk levels when compared to using BRI alone. Karvonen-Gutierrez et al. observed similar phenomena, finding that despite similar AUCs for different obesity measurement methods, they might exhibit significant differences in risk classification capability (36). This underscores the importance of utilizing multiple statistical approaches when evaluating prediction models, rather than relying solely on discrimination metrics such as AUC. When comparing the combined model to METS-VF alone, the improvement was much more limited, with a small NRI of 0.003 (95%CI: 0.001-0.005, P = 0.004) and a non-significant IDI of 0.076 (95%CI: -0.026-0.174, P = 0.156). This suggests that METS-VF, as a comprehensive metabolic indicator, may already capture most of the information relevant to OA risk, with BRI providing minimal additional predictive value beyond what METS-VF already contributes. This finding aligns with Hart et al.’s research comparing different metabolic indicators, which found that comprehensive metabolic indicators generally have better predictive ability than single body shape indicators (37). These results suggest that the clinical value of combining BRI and METS-VF depends on the specific context and purpose. For practices currently using BRI alone, adding METS-VF assessment would provide significant improvement in risk classification. However, for those already using METS-VF, there would be limited benefit in additionally measuring BRI.

Our research found that patients with a combination of high BRI (≥5.865) and low METS-VF (<7.255) might have the highest risk of OA, although this association did not reach statistical significance. This finding differs somewhat from traditional views, which suggest that the synergistic effect of metabolic abnormalities and obesity should lead to the highest OA risk (38). Our results may reflect the so-called “metabolic phenotype diversity,” where different metabolic phenotypes might influence OA risk through various mechanisms. Zhuo et al. proposed that the special phenotypes of “metabolically healthy obesity” and “metabolically unhealthy non-obesity” might affect OA onset and progression (13). The high BRI and low METS-VF combination we observed may represent a unique metabolic phenotype, where abnormal body shape distribution (central obesity) coexists with relatively good metabolic parameters. Richmond et al.’s research supports this view, finding that the associations between certain metabolic indicators and OA might vary depending on body type characteristics (39). This suggests that when assessing OA risk, we should not simply assume an additive effect of metabolic abnormalities and obesity, but rather consider the specificity of different metabolic phenotypes.

Subgroup analysis results showed that the associations of BRI and METS-VF with OA risk remained consistent across different age, gender, race, and hypertension status subgroups, with no statistically significant interactions (P>0.05). This consistency enhances the reliability and universality of our main findings, indicating that the roles of METS-VF and BRI as predictors of OA risk are relatively robust and not significantly affected by major demographic characteristics. This result aligns with Felson et al.’s multicenter study findings, which showed that the impact of metabolic factors on OA risk was relatively stable across populations with different demographic characteristics (40). However, contrary to our findings, Sowers et al. reported that the influence of metabolic syndrome on OA risk might be greater in women than in men (41). Lee et al.’s study also found that the association between visceral fat and OA might be particularly strong in postmenopausal women (42). These differences may stem from variations in study design, sample size, OA definition methods, and adjusted confounding factors.

Nevertheless, this study has several limitations that need to be considered. Firstly, the cross-sectional design limits causal inference, making it impossible to determine whether elevated METS-VF and BRI are causes or consequences of OA, or whether both are influenced by common pathophysiological mechanisms; this requires further verification through prospective cohort studies. Secondly, OA diagnosis was based on self-reported physician diagnosis rather than clinical examination or imaging confirmation, which may lead to a certain degree of misclassification. Thirdly, although we adjusted for many known confounding factors, the possibility of residual confounding cannot be ruled out. For example, we lack information on joint injury history, occupation-related risk factors, and medication use (such as glucocorticoids), which might influence OA risk. Finally, we did not distinguish between OA in different joint sites (such as knee, hip, or hand OA), while OA at different sites might be affected by metabolic factors to varying degrees.

## 5 Conclusions

In conclusion, this study demonstrates that METS-VF and BRI are independently associated with OA in patients with diabetes or prediabetes, with both showing significant dose-response relationships. These findings emphasize the important roles of metabolic health and body shape distribution in their relationship with OA among patients with diabetes/prediabetes, providing new biomarkers for clinical assessment and potential targets for intervention. Future research should explore the associations of these indicators across different OA subtypes and validate their potential predictive value through prospective study designs.

## Data Availability

The original contributions presented in the study are included in the article/supplementary material. Further inquiries can be directed to the corresponding authors.

## References

[1] Martel-Pelletier J Barr AJ Cicuttini FM Conaghan PG Cooper C Goldring MB . Osteoarthritis. Nat Rev Dis primers. (2016) 2:16072. doi: 10.1038/nrdp.2016.72, PMID: 27734845

[2] Hunter DJ Bierma-Zeinstra S . Osteoarthritis. Lancet. (2019) 393:1745–59. doi: 10.1016/S0140-6736(19)30417-9, PMID: 31034380

[3] Loeser RF Goldring SR Scanzello CR Goldring MB . Osteoarthritis: a disease of the joint as an organ. Arthritis Rheumatol. (2012) 64:1697–707. doi: 10.1002/art.34453, PMID: 22392533 PMC3366018

[4] GBD 2017 Disease and Injury Incidence and Prevalence Collaborators . Global, regional, and national incidence, prevalence, and years lived with disability for 354 diseases and injuries for 195 countries and territories, 1990-2017: a systematic analysis for the Global Burden of Disease Study 2017. Lancet. (2018) 392(10159):1789–858. doi: 10.1016/S0140-6736(18)32279-7, PMID: 30496104 PMC6227754

[5] Pettenuzzo S Berardo A Belluzzi E Pozzuoli A Ruggieri P Carniel EL . Mechanical insights into fat pads: a comparative study of infrapatellar and suprapatellar fat pads in osteoarthritis. Connect Tissue Res. (2025) 66:272–83. doi: 10.1080/03008207.2025.2502591, PMID: 40340764

[6] Klein-Wieringa IR Kloppenburg M Bastiaansen-Jenniskens YM Yusuf E Kwekkeboom JC El-Bannoudi H . The infrapatellar fat pad of patients with osteoarthritis has an inflammatory phenotype. Ann Rheum Dis. (2011) 70:851–7. doi: 10.1136/ard.2010.140046, PMID: 21242232

[7] Clockaerts S Bastiaansen-Jenniskens YM Runhaar J Van Osch GJ Van Offel JF Verhaar JA . The infrapatellar fat pad should be considered as an active osteoarthritic joint tissue: a narrative review. Osteoarthritis Cartilage. (2010) 18:876–82. doi: 10.1016/j.joca.2010.03.014, PMID: 20417297

[8] Bastiaansen-Jenniskens YM Clockaerts S Feijt C Zuurmond AM Stojanovic-Susulic V Bridts C . Infrapatellar fat pad of patients with end-stage osteoarthritis inhibits catabolic mediators in cartilage. Ann Rheum Dis. (2012) 71:288–94. doi: 10.1136/ard.2011.153858, PMID: 21998115

[9] Allen KD Thoma LM Golightly YM . Epidemiology of osteoarthritis. Osteoarthritis Cartilage. (2022) 30:184–95. doi: 10.1016/j.joca.2021.04.020, PMID: 34534661 PMC10735233

[10] Palazzo C Nguyen C Lefevre-Colau MM Rannou F Poiraudeau S . Risk factors and burden of osteoarthritis. Ann Phys Rehabil Med. (2016) 59:134–8. doi: 10.1016/j.rehab.2016.01.006, PMID: 26904959

[11] Berenbaum F . Diabetes-induced osteoarthritis: from a new paradigm to a new phenotype. Postgraduate Med J. (2012) 88:240–2. doi: 10.1136/pgmj.2010.146399rep, PMID: 22441236

[12] Williams MF London DA Husni EM Navaneethan S Kashyap SR . Type 2 diabetes and osteoarthritis: a systematic review and meta-analysis. J Diabetes its complications. (2016) 30:944–50. doi: 10.1016/j.jdiacomp.2016.02.016, PMID: 27114387

[13] Zhuo Q Yang W Chen J Wang Y . Metabolic syndrome meets osteoarthritis. Nat Rev Rheumatol. (2012) 8:729–37. doi: 10.1038/nrrheum.2012.135, PMID: 22907293

[14] Tchernof A Després JP . Pathophysiology of human visceral obesity: an update. Physiol Rev. (2013) 93:359–404. doi: 10.1152/physrev.00033.2011, PMID: 23303913

[15] Huang TD Behary J Zekry A . Non-alcoholic fatty liver disease: a review of epidemiology, risk factors, diagnosis and management. Internal Med J. (2020) 50:1038–47. doi: 10.1111/imj.14709, PMID: 31760676

[16] Kahn HS Cheng YJ . Comparison of adiposity indicators associated with fasting-state insulinemia, triglyceridemia, and related risk biomarkers in a nationally representative, adult population. Diabetes Res Clin practice. (2018) 136:7–15. doi: 10.1016/j.diabres.2017.11.019, PMID: 29183845 PMC6003239

[17] Thomas EL Frost G Taylor-Robinson SD Bell JD . Excess body fat in obese and normal-weight subjects. Nutr Res Rev. (2012) 25:150–61. doi: 10.1017/S0954422412000054, PMID: 22625426

[18] Thomas DM Bredlau C Bosy-Westphal A Mueller M Shen W Gallagher D . Relationships between body roundness with body fat and visceral adipose tissue emerging from a new geometrical model. Obes (Silver Spring Md). (2013) 21:2264–71. doi: 10.1002/oby.20408, PMID: 23519954 PMC3692604

[19] Chang Y Guo X Chen Y Guo L Li Z Yu S . A body shape index and body roundness index: two new body indices to identify diabetes mellitus among rural populations in northeast China. BMC Public Health. (2015) 15:794. doi: 10.1186/s12889-015-2150-2, PMID: 26286520 PMC4544789

[20] Francisco V Pérez T Pino J López V Franco E Alonso A . Biomechanics, obesity, and osteoarthritis. The role of adipokines: When the levee breaks. J orthopaedic Res. (2018) 36:594–604. doi: 10.1002/jor.23788, PMID: 29080354

[21] Veronese N Cooper C Reginster JY Hochberg M Branco J Bruyère O . Type 2 diabetes mellitus and osteoarthritis. Semin Arthritis rheumatism. (2019) 49:9–19. doi: 10.1016/j.semarthrit.2019.01.005, PMID: 30712918 PMC6642878

[22] Zhang W Likhodii S Zhang Y Aref-Eshghi E Harper PE Randell E . Classification of osteoarthritis phenotypes by metabolomics analysis. BMJ Open. (2014) 4:e006286. doi: 10.1136/bmjopen-2014-006286, PMID: 25410606 PMC4244434

[23] Mobasheri A Rayman MP Gualillo O Sellam J van der Kraan P Fearon U . The role of metabolism in the pathogenesis of osteoarthritis. Nat Rev Rheumatol. (2017) 13:302–11. doi: 10.1038/nrrheum.2017.50, PMID: 28381830

[24] American Diabetes Association Professional Practice Committee . 2. Diagnosis and classification of diabetes: standards of care in diabetes-2024. Diabetes Care. (2024) 47:S20–42., PMID: 38078589 10.2337/dc24-S002 PMC10725812

[25] He Q Wang Z Mei J Xie C Sun X . Relationship between systemic immune-inflammation index and osteoarthritis: a cross-sectional study from the NHANES 2005-2018. Front Med. (2024) 11:1433846. doi: 10.3389/fmed.2024.1433846, PMID: 39206165 PMC11349521

[26] Xue H Zhang L Xu J Gao K Zhang C Jiang L . Association of the visceral fat metabolic score with osteoarthritis risk: a cross-sectional study from NHANES 2009-2018. BMC Public Health. (2024) 24:2269. doi: 10.1186/s12889-024-19722-0, PMID: 39169311 PMC11337595

[27] Niu J Clancy M Aliabadi P Vasan R Felson DT . Metabolic syndrome, its components, and knee osteoarthritis: the Framingham osteoarthritis study. Arthritis Rheumatol (Hoboken NJ). (2017) 69:1194–203. doi: 10.1002/art.40087, PMID: 28257604 PMC5449217

[28] Lohmander LS Gerhardsson de Verdier M Rollof J Nilsson PM Engström G . Incidence of severe knee and hip osteoarthritis in relation to different measures of body mass: a population-based prospective cohort study. Ann rheumatic diseases. (2009) 68:490–6. doi: 10.1136/ard.2008.089748, PMID: 18467514

[29] Rico-Martín S Calderón-García JF Sánchez-Rey P Franco-Antonio C Martínez Alvarez M Sánchez Muñoz-Torrero JF . Effectiveness of body roundness index in predicting metabolic syndrome: A systematic review and meta-analysis. Obes Rev. (2020) 21:e13023. doi: 10.1111/obr.13023, PMID: 32267621

[30] Collins KH Herzog W MacDonald GZ Reimer RA Rios JL Smith IC . Obesity, metabolic syndrome, and musculoskeletal disease: common inflammatory pathways suggest a central role for loss of muscle integrity. Front Physiol. (2018) 9:112. doi: 10.3389/fphys.2018.00112, PMID: 29527173 PMC5829464

[31] Schett G Kleyer A Perricone C Sahinbegovic E Iagnocco A Zwerina J . Diabetes is an independent predictor for severe osteoarthritis: results from a longitudinal cohort study. Diabetes Care. (2013) 36:403–9. doi: 10.2337/dc12-0924, PMID: 23002084 PMC3554306

[32] Rosa SC Gonçalves J Judas F Mobasheri A Lopes C Mendes AF . Impaired glucose transporter-1 degradation and increased glucose transport and oxidative stress in response to high glucose in chondrocytes from osteoarthritic versus normal human cartilage. Arthritis Res Ther. (2009) 11:R80. doi: 10.1186/ar2713, PMID: 19490621 PMC2714130

[33] Dawson LP Fairley JL Papandony MC Hussain SM Cicuttini FM Wluka AE . Is abnormal glucose tolerance or diabetes a risk factor for knee, hip, or hand osteoarthritis? A systematic review. Semin Arthritis rheumatism. (2018) 48:176–89. doi: 10.1016/j.semarthrit.2018.02.008, PMID: 29550110

[34] Cheon YH Kim HO Suh YS Kim MG Yoo WH Kim RB . Relationship between decreased lower extremity muscle mass and knee pain severity in both the general population and patients with knee osteoarthritis: Findings from the KNHANES V 1-2. PloS One. (2017) 12:e0173036. doi: 10.1371/journal.pone.0173036, PMID: 28296926 PMC5351834

[35] Berenbaum F Griffin TM Liu-Bryan R . Review: metabolic regulation of inflammation in osteoarthritis. Arthritis Rheumatol (Hoboken NJ). (2017) 69:9–21. doi: 10.1002/art.39842, PMID: 27564539 PMC5341385

[36] Karvonen-Gutierrez CA Harlow SD Jacobson J Mancuso P Jiang Y . The relationship between longitudinal serum leptin measures and measures of magnetic resonance imaging-assessed knee joint damage in a population of mid-life women. Ann rheumatic diseases. (2014) 73:883–9. doi: 10.1136/annrheumdis-2012-202685, PMID: 23576710 PMC3884071

[37] Hart DJ Doyle DV Spector TD . Association between metabolic factors and knee osteoarthritis in women: the Chingford Study. J Rheumatol. (1995) 22:1118–23. 7674240

[38] Yucesoy B Charles LE Baker B Burchfiel CM . Occupational and genetic risk factors for osteoarthritis: a review. Work (Reading Mass). (2015) 50:261–73. doi: 10.3233/WOR-131739, PMID: 24004806 PMC4562436

[39] Richmond SA Fukuchi RK Ezzat A Schneider K Schneider G Emery CA . Are joint injury, sport activity, physical activity, obesity, or occupational activities predictors for osteoarthritis? A systematic review. J orthopaedic sports Phys Ther. (2013) 43:515–b19. doi: 10.2519/jospt.2013.4796, PMID: 23756344

[40] Felson DT Zhang Y Anthony JM Naimark A Anderson JJ . Weight loss reduces the risk for symptomatic knee osteoarthritis in women. Framingham Study. Ann Internal Med. (1992) 116:535–9. doi: 10.7326/0003-4819-116-7-535, PMID: 1543306

[41] Sowers M Karvonen-Gutierrez CA Palmieri-Smith R Jacobson JA Jiang Y Ashton-Miller JA . Knee osteoarthritis in obese women with cardiometabolic clustering. Arthritis rheumatism. (2009) 61:1328–36. doi: 10.1002/art.24739, PMID: 19790111 PMC2776774

[42] Lee S Kim TN Kim SH . Sarcopenic obesity is more closely associated with knee osteoarthritis than is nonsarcopenic obesity: a cross-sectional study. Arthritis rheumatism. (2012) 64:3947–54. doi: 10.1002/art.37696, PMID: 23192792

